# Conservative management of dentigerous cysts in children

**DOI:** 10.4317/jced.52248

**Published:** 2015-12-01

**Authors:** Manuel Arjona-Amo, María-Angeles Serrera-Figallo, José-María Hernández-Guisado, José-Luis Gutiérrez-Pérez, Daniel Torres-Lagares

**Affiliations:** 1 Department of Stomatology, University of Seville, 41009, Seville, Spain; 2 Virgen del Rocío Hospital, Clinical Management Unit (UGC) Oral and Maxillofacial Surgery, Seville, Spain

## Abstract

**Purpose and Introduction:**

Dentigerous cysts are epithelial in origin and are the most commonly found cyst in children. The majority of these lesions are usually a radiological finding and are capable of quite large before being diagnosed. The standard treatment for these cysts is the enucleation and the extraction of the affected tooth. However, if the patient is a child and the affected tooth is not developed, a more conservative attitude should be considered.

**Material and Methods:**

(Clinical case): A 7-year-old patient is presented with an eruptive backlog of the lower permanent first molars. Radiological examination reveals two radiolucid lesions in relation to them, which are compatible with a dentigerous cyst, and in relation to the inferior aveolar nerve and various germs. A partial enucleation is carried out, maintaining all the dental germs related to the cyst in mouth and monitoring the patient until the case study is over.

**Results and Discussion:**

Diagnosis and early treatment of these lesions in children is of great importance, especially in cases where the lesions enclose permanent teeth.

**Conclusions:**

Whenever possible, a conservative attitude should be taken, one that allows for the maintenance of the dentition and treatment of the associated cyst in order to not compromise either the occlusion or the mental state of these patients.

** Key words:**Dentigerous cyst, conservative treatment, dental impaction, child.

## Introduction

Dentigerous cysts are the second most common cystic lesion to affect the mandibular. They account for 14-20% of mandibular cysts and between 15.2% and 33.7% of all odontogenic cysts (1). The frequency with which dentigerous cysts develop has been calculated at 1.44 in every 100 unerupted teeth (1). They are more frequent in men than in women and are more frequent in Caucasians than in individuals with darker skin (1).

Dentigerous cysts derive from a change in the development of the reduced epithelium enamel organ, which results in an accumulation of fluid between this and the permanent tooth’s crown (2). There are two theories to explain the association of these cysts with the lower primary second molars. The first is that the second molar is more susceptible to caries. The second is that the germ of the primary second molar is closer to the permanent premolar (3).

Dentigerous cysts are typically asymptomatic and are an incidental finding on routine radiographs. They are rarely painful and any pain suffered is associated with infection in the lesion (4). In some instances, these cysts can grow to very large size and can trigger the inflammation, expansion and erosion of the cortical bone. In such a case, they can generate a differential diagnosis to an ameloblastoma or an odontogenic keratocystic tumour. On radiographs, dentigerous cysts appear as a radiotransparent, round, well-defined image that is in close relation to the crown of an enclosed tooth (5).

With respect to the above approach, different treatment options exist for these lesions. The classic approach is the removal of the cyst together with the affected tooth, to allow the regeneration of healthy bone. At times, this approach is too aggressive and more conservative ones (such as decompression, marsupialization, etc.) should be considered, as they can be advantageous in the sense that they allow for the eruption of the teeth related to the cyst (6).

At all times, cases studies should be assessed on an individual basis when deciding on the treatment method that is most appropriate for the size and location of the cyst, the age of the patient, the affected dentition and the commitment to noble structures.

## Case Study

**Figure 1:**
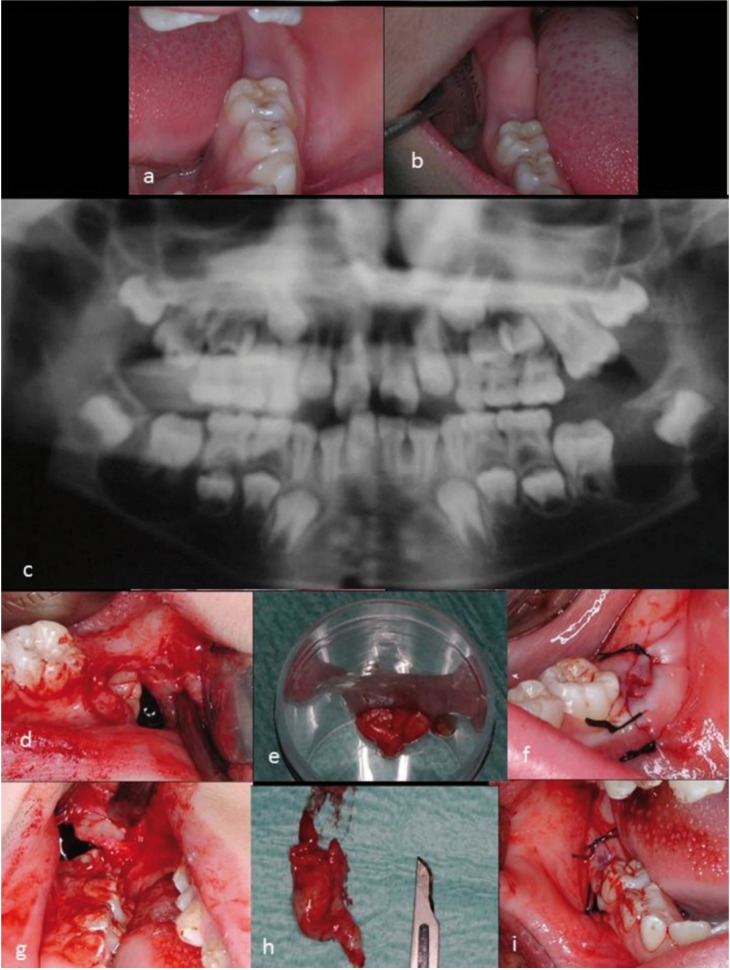
a) Intraoral image, section 30. b) Intraoral image, section 40. c) Panoramic radiograph that shows the presence of radioludid lesions in relation to the first permanent mandibular molars. D to I: Surgical intervention on cystic lesions. d) Opening of the cystic cavity in section 30, an enclosed tooth is revealed. e) Surgical specimen for anatompatholigal study. f) Non-watertight suture. g) Opening of the cystic cavity in section 40, an enclosed tooth is revealed. h) Surgical specimen for anatompatholigal study. i) Non-watertight suture.

A 7-year-old boy is referred to the Oral and Maxilofacial Surgery Department, Virgen del Rocío Hospital in Seville. Intraoral examination revealed that the patient presented a mixed dentition with an eruptive backlog (Fig. 1a,b).

On the orthopantomograph, two compatible radiolucid areas with cystic cavities are clearly visible, in close proximity to the lower permanent first molars, which is triggering impaction (Fig. 1c).

A well-defined radiolucid lesion was noted in relation to tooth 36, which encloses it completely. It rejects the inferior alveolar nerve all the way to the mandibular basal bone and affects the germ of the second molar, displacing it distally.

The lesion that affects tooth 46 completely encloses it and expands towards the mandibular basal bone displacing the inferior alveolar nerve. It also affects germ of tooth 47. In both situations, it is possible that the germ of the permanent second molar is affected.

Various types of lesions are included in the differential diagnosis (unicystic ameloblastoma, dentigerous cyst, keratocystic odon-togenic tumour, etc.). In all cases studies, anatompatholigal study is necessary to arrive at the correct diagnosis.

When looking at clinical findings, presumptions are made about dentigerous cysts. Treatment should comprise the removal of both lesions, which would entail the removal of both first permanent molars, possibly the second permanent molars and second permanent premolars. Careful dissection of the inferior alveolar nerve should be carried out.

The possibility of bone fracture and the safe change in the patient’s mandibular growth should also be assessed.

For this reason, more conservative and less traditional options should be explored. The success of these techniques is put down to maintaining eruptive potential of the two lower permanent first molars that are still yet to conclude their development.

After signing informed consent, the first lesion that was treated was located on tooth 36, where a partial removal (and subsequent anatomapathologial study) of its capsule and non-watertight suture was carried out, without at any point either touching tooth 36 or approaching inferior alveolar nerve (Fig. 1d-f). A month later, the same procedure was repeated on the lesion located on tooth 46 (Fig. 1g-i). In both cases, the anatomopathological report identified the lesion as a dentigerous cyst.

**Figure 2:**
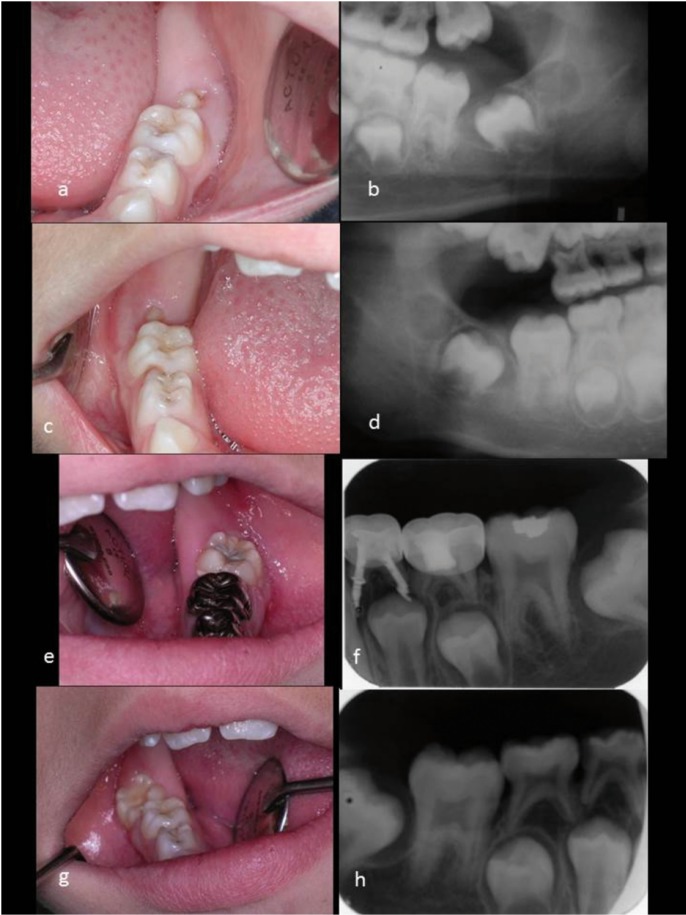
A to D: Follow up, three months after the first surgical intervention. a) Intraoral image section 30. b) Radiograph of section 30. c) Intraoral image section 40. d) Radiograph of section 40. E to H: Follow up, two years after the first surgical intervention. e) Intraoral image section 30. f) Radiograph of section 30. g) Intraoral image section 40. h) Radiograph of section 40.

Three months after the procedure, the first permanent molars erupted (Fig. 2a-d). In the routine follow ups, it was noted that the permanent first molar occupied its position in the arch and that the cystic cavity disappeared completely.

In the one and two year follow ups (Fig. 2e-h), it was noted that the eruptive process continued normally. Only a slight misalignment, caused by the appearance of both cysts, of the permanent second molars was noted. This could be resolved by your orthodontist. Molars are essential and their development completely normal.

## Discussion

Dentigerous cysts are odontogenic cysts that typically affect impacted teeth, in the majority of cases mandibular third molars and maxillary canines (7,8). However, the case study in question refers to a primary first molar at a stage of formation, which is an uncommon situation.

This case also presents other aspects that should be considered and which complicate the approach: various permanent germs and the inferior alveolar nerve are affected, as well as the possibility of pathologic fracture if a radical approach is carried out, etc.

Various therapeutic options are also detailed with respect to the management of dentigerous cysts in children, which include the complete enucleation of these lesions with exodontia of the affected teeth, as well as other conservative options such as marsupialization, decompression either with or without traction of the tooth to its correct position in the arch.

Now that the problems relating to cystic enucleation have been discussed, it is believed that, in these instances, other, more con-servative that minimise the harm to the patient should be considered.

Yahara (9) and Hyomoto (10) have found that between 71.4 and 72.4% of the individuals who participated in their study presented with natural eruption of teeth enclosed in the cyst after having carried out marsupialization.

Muramaki *et al.* (11) published a case study about a dentigerous cyst belonging to a boy of twelve years, which was located on the same level as the lower left second premolar and which was treated through marsupialization.

Berti *et al.* (12) present a case study about a dentigerous cyst with repeated episodes of infection related to the mandibular canine, a deciduous primary molar, a mandibular canine and the first permanent premolar, which triggered the impaction of these teeth. In this case, after administering antibiotics and carrying out exodontia of the canine and the deciduous primary molar, marsupialization was conducted to try and save the implicated permanent teeth and after 12 months, a complete reduction of the lesion and the eruption of the canine and first premolar was noted.

Sight should also not be lost of those case studies where cysts have started to develop. In said case studies, a clear natural communication between the enclosed tooth and the oral cavity was noted. In some instances, this is the approved method of decompresion (13).

In this instance, the cystic lesion and the occlusal surfaces of the first molars were located very close to the oral mucosa. For this reason, it is believed that partial removal of the cystic capsule together with a non-watertight suture of the wound could give the opportunity to establish this communication, i.e.: (the eruption of the molars took place quickly as a result of the reduction of the cystic pressure exerted on them).

For Hyomoto, (10) the key factors in the eruption of the tooth in the arch are bone development, the angle and the depth of the tooth in the maxillary. The impacted teeth together with incomplete root development show potential for eruption, which takes place when they have two thirds of the root formation emerging in the oral cavity when they have approximately three quarters of root formation. It has also been proven that those teeth that present an axial angle less than 80º and a depth in the maxillary less than 9 mm have significantly higher chances of erupting. The size of the cyst and the existing space between adjacent teeth appear to have no influence on the eruption of these teeth.

Equally, Fujii *et al.* (14) indicated that the probability of eruption of a permanent tooth enclosed in a dentigerous cyst increases in patients over the age of 10, in those where their tooth has a depth less than 5.1 mm and where the angle of the tooth is less than 25º and the space between adjacent teeth was greater than the size of the teeth. Such factures, indicated by Hyomoto and Fujii *et al.* apply to this case study.

Yahara (9) and Hyomoto (10) establish that the average time it takes teeth to erupt without carrying out orthodontic traction is approximately three months in comparison to a period of some hundred days after the more conservative approach has been used to decide whether to extract or carry out orthodontic traction. In this case study, the teeth erupted within the three month period.

## Conclusion

The diagnosis and early treatment of lesions in children, such as those presented in this article, is of great importance above all in case studies where lesions that enclose permanent teeth in order to minimize the associated damages to what is done.

We should always study, and where possible opt for, a conservative attitude that allows for the maintenance of the dentition and treatment of the associated cyst in order to not compromise either the occlusion or the mental state of these patients.
